# Rapid quantitative analysis of potassium in soil based on direct-focused laser ablation-laser induced breakdown spectroscopy

**DOI:** 10.3389/fchem.2022.967158

**Published:** 2022-09-01

**Authors:** Peng-Cheng Han, Kun Yang, Lei-Zi Jiao, Hua-Chang Li

**Affiliations:** ^1^ Department of Chemistry and Chemical Engineering, University of Science and Technology Beijing, Beijing, China; ^2^ BGRIMM Technology Group, Beijing, China; ^3^ Research Center of Intelligent Equipment, Beijing Academy of Agriculture and Forestry Sciences, Beijing, China

**Keywords:** LIBS, potassium in soil, matrix effect, laser ablation-assisted, field

## Abstract

A fast quantitative analysis method of soil potassium based on direct-focused laser ablation-laser induced breakdown spectroscopy (direct-focused LA-LIBS) was proposed and tested. A high single-pulse energy laser (200 mJ/pulse) beam was focused on the aerosols near the focus of the 10 kHz fiber laser to generate plasma spectra, and the analytical capability of the direct-focused LA-LIBS system was compared with traditional LIBS system using a high single-pulse energy laser (SP-LIBS). The result showed that for moist soil samples the data stability of the direct-focused LA-LIBS method was significantly improved and the R^2^ factor of the calibration curve improved from 0.64 to 0.93, the limit of detection improved from 159.2 μg/g to 140.9 μg/g. Three random soil samples from different areas of Beijing suburbs were analyzed by the direct-focused LA-LIBS method, and the results were consistent with AAS. The direct-focused LA-LIBS method proposed is different from the traditional double-pulse technology and laser ablation-assisted technology because it not only does not need carrier gas, but also can overcome the matrix differences better, especially the influence of moisture, which provides a new idea for the rapid detection of nutrient elements in field soils.

## 1 Introduction

Rapid detection of soil nutrients has been the focus of research worldwide (Hussain et al., 2007; Gholizadeh et al., 2013). The potassium content is an important indicator of soil fertility (Dale, 1975; Assefa and Ledin, 2001; Mohammad et al., 2003), and is the most important trace element for plants (Toader et al., 2013; Wang et al., 2013). Rapid detection of potassium in soil provides guidance for rational fertilization and basis data for agricultural farmland census and production planning (Chen et al., 2013; Nikkhoo et al., 2013; Zhang et al., 2015; Fu et al., 2020).

Current methods to detect potassium in soil are based on laboratory analysis, such as inductively coupled plasma spectrometry (KOVACS, 2000), atomic absorption spectrometry (David, 1960), or titration (Haney et al., 2008), which are highly accurate, but inflexible and cannot meet the needs of large scale field detection (Jackson, 2015). Laser-induced breakdown spectroscopy (LIBS) is a popular, rapid detection technique with non-contact and real-time analysis (Fortes et al., 2013; Bol'Shakov et al., 2010), and many studies have used it for the detection of potassium in soil (Hussain and Gondal, 2008; Burakov et al., 2010; Khoso et al., 2021).

With different soil types and matrices, the accuracy of the analytical results may be affected (Bousquet et al., 2007; Bae, 2015; Nicolodelli et al., 2015), and therefore post-processing by algorithms is used to eliminate them (Li et al., 2020; Liu et al., 2022). Lu et al. designed an analytical model for potassium in soil based on convolutional neural networks (CNNs) with an R^2^ factor of 0.99 on the calibration curve, demonstrating that the LIBS technique can reduce the matrix effects through the algorithms (Chengxu et al., 2018). Yong He et al. used a combination of double-pulse LIBS technique and a partial least squares (PLSR) algorithm to analyze nutrients in 63 different soil samples and demonstrated that the PLSR could effectively improve the detection limit and analytical accuracy of the model (He et al., 2018). The drawback of this algorithm-based approach is how to find a sufficient number of representative samples, a difficult task for field soil (Islam and Chowdhury, 2002; Yang et al., 2015).

Recent studies have focused on reducing the interference of matrix effects by starting from the characteristics and production process of plasma. For example, Ciucci et al. proposed a method with calibration-free analysis (CF-LIBS). This was based on the relationship between spectral intensity, atomic transition energy, plasma temperature, and electron density, which do not need to establish a calibration curve based on standard samples, so the influence of the matrix can theoretically be avoided (CiucciCorsi et al., 1999). However, this method is very difficult to be applied due to the calculation of plasma temperature, electron density, the elimination of self-absorption effects, and the acquisition of the total element spectrum (Tognoni et al., 2009; Elsayed et al., 2022).

Another approach to reduce matrix effect is to separate the two processes of laser-ablation and plasmaization. The aerosol produced by laser ablation is transported to a new cavity through a carrier gas, and then the plasma is excited, collected, and analyzed. Hahn et al. investigated SP-LA-LIBS, DP-LA-LIBS, and LA-LIBS systems (Windom and Hahn, 2009; Pareja et al., 2013; Glaus and Hahn, 2014; Wang et al., 2021) and showed that the LA-LIBS systems, which require a carrier gas for transmission, can significantly reduce the matrix effect, but the whole system structure is complex, and the limit of detection is high. In addition, the influence of soil moisture on the spectral signal should not be neglected in the field analysis, but there is a lack of relevant studies using traditional LA-LIBS.

In order to improve the field analysis capability of LA-LIBS, this study tested a rapid analysis method by focusing a high single-pulse energy laser directly on the aerosol near the 10 kHz fiber laser focus point (direct-focused LA-LIBS) and compared the analytical performance with conventional LIBS system using one laser with high single-pulse energy (SP-LIBS). We demonstrate that the direct-focused LA-LIBS method can eliminate the effects of differences in soil type and water content within a limited range. This is the first successful study using LA-LIBS without a sample chamber and carrier gas to analyze soils, which will give a good impetus to the practical application of LIBS technology in the field.

## 2 Materials and methods

### 2.1 Experimental materials

A total of eight national standard soil samples were used in this experiment. The sample numbers and soil properties are shown in Table 1.

**TABLE 1:** Sample labels, locations, and properties used in the experiment.

	Number	Sampling locations	Properties of the sample	Potassium content (%)
1	GBW07401a	Yichun, Heilongjiang	Soil in Xilin Lead-zinc mining area	2.85
2	GBW07402a	Bayan Obo, Inner Mongolia	Soil in rare earth mining area of Bayan Obo Iron Mine	3.0
3	GBW07403a	Laizhou, Shandong	Soil in around the gold mine	2.9
4	GBW07404a	Yizhou, Guangxi	Soil in limestone area	3.0
5	GBW07405a	Liuyang, Hunan	Soil in Qibaoshan polymetallic mining area	2.1
6	GBW07406a	Yangchun, Guangdong	Soil in Yangchun Xishan tungsten tin polymetallic mining area	0.44
7	GBW07407a	Xuwen, Guangdong	Soil in Leizhou Peninsula	0.35
8	GBW07408a	Luochuan, Shanxi	Soil in Loess plateau	2.3

These standard soil samples come from Northeast, North, Northwest, and Southeast China, with diverse soil types and strong representativeness.

1) making the first group of soil samples for the calibration of the characteristic spectral lines of potassium: three standard soil samples with the same weight (GBW07407A, 30g/piece) were mixed with three different contents of potassium chloride aqueous solution samples, and after air drying, grinding and tablet pressing, three calibration samples (sample1,sample2,sample3) of the same matrix type are formed, and the content of potassium in sample1 is 0.35%, in sample2 is 2.0%, in sample3 is 3.7%. The relevant characteristics of the first group of soil samples are shown in Table 2.

**TABLE 2:** Relevant characteristics of the first group of tablet samples.

Number	Corresponding standard sample	Potassium content (%)	Water content
Sample1	GBW07407a	0.35	dry
Sample2	GBW07407a	2.0	
Sample3	GBW07407a	3.7	

2) Eight dry soil tablets were made as the second group. Take 30 g of soil from each of the eight standard soil samples, and then put them respectively into an aluminum box with a diameter of 40 mm and pressed into soil tablets, so eight kinds of dry soil tablets were formed: sample4 to sample11. The relevant characteristics of the second group of soil samples are shown in Table 3.

**TABLE 3:** Relevant characteristics of the second group of tablet samples.

Number	Corresponding standard sample	Potassium content (%)	Water content
Sample4	GBW07401a	2.85	dry
Sample5	GBW07402a	3.0	
Sample6	GBW07403a	2.91	
Sample7	GBW07404a	3.0	
Sample8	GBW07405a	2.1	
Sample9	GBW07406a	0.44	
Sample10	GBW07407a	0.35	
Sample11	GBW07408a	2.3	

3) Eight moist soil tablets were made as the third group. Similarly, 30 g of soil were also taken from each of the eight standard samples and placed into an aluminum box respectively with a diameter of 40 mm 1, 2, 3, 4, 5, 6, 7 and 8 ml of pure water were added to the aluminum box in turn. After stirring, the soil in the aluminum box was pressed into a set of soil samples with different water contents, so eight kinds of moist soil tablets were formed: sample12 to sample19. The relevant characteristics of the third group of soil samples are shown in Table 4.

**TABLE 4:** Relevant characteristics of the third group of tablet samples.

Number	Corresponding standard sample	Potassium content (%)	Water content (%)
Sample12	GBW07401a	2.85	3.2
Sample13	GBW07402a	3.0	6.3
Sample14	GBW07403a	2.9	9.1
Sample15	GBW07404a	3.0	11.8
Sample16	GBW07405a	2.1	14.3
Sample17	GBW07406a	0.44	16.7
Sample18	GBW07407a	0.35	18.9
Sample19	GBW07408a	2.3	21.1

### 2.2 Experimental system

**FIGURE 1:**
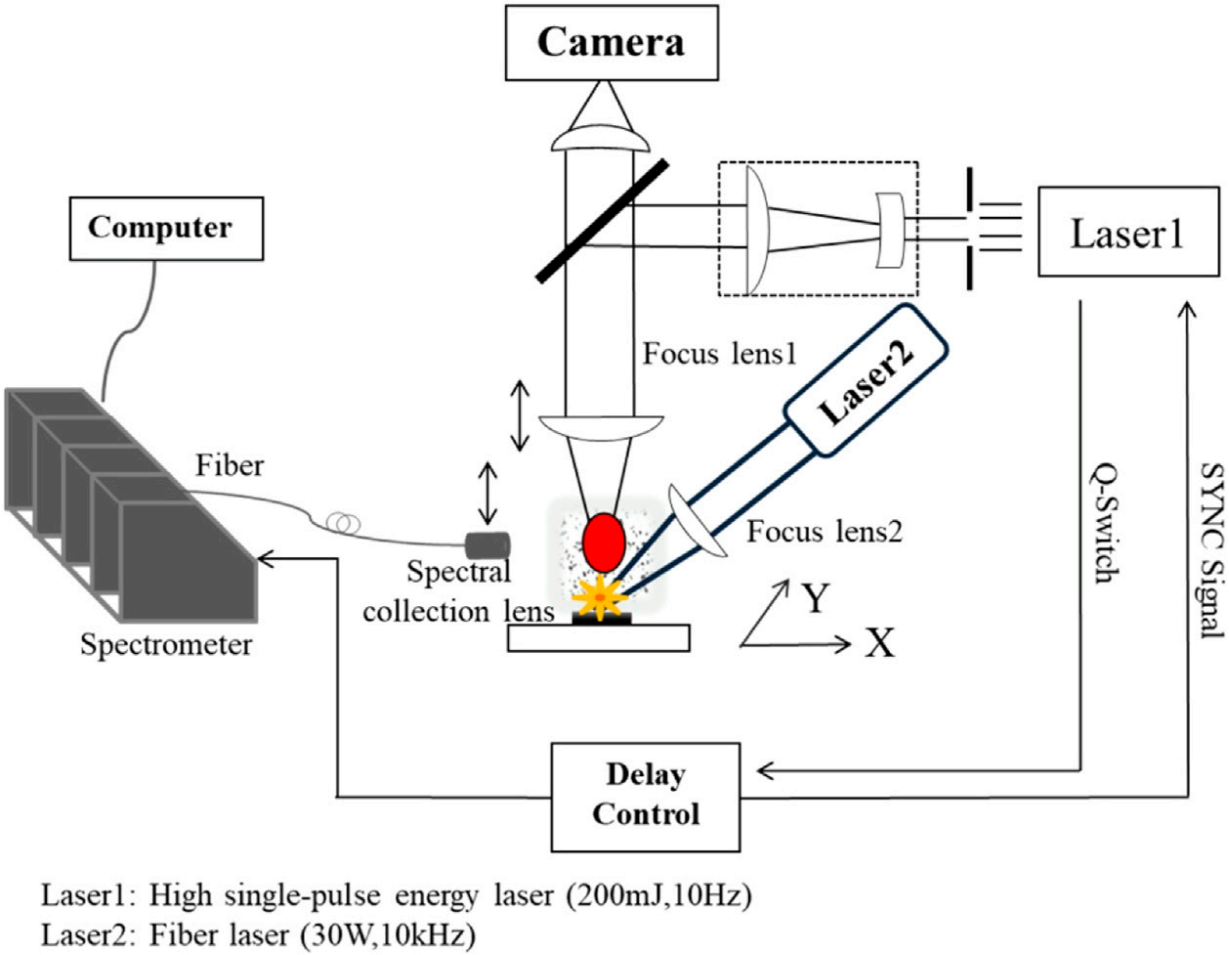
System structure diagram.

The experimental system is shown in Figure 1. There were two lasers in the system, one of which is a high single-pulse energy laser named Tiny200 series laser produced by Grace Laser Technology Co., Ltd (1,064 nm, 200 mJ/pulse, 10 Hz, 8 ns pulse-width), additionally a fiber laser with a wavelength of 1080 nm produced by Taizhou laser Co., Ltd. was customized. The maximum frequency of our customized fiber laser can reach 10 kHz, and the single pulse energy can reach 3 mJ/pulse, which is basically the upper limit of the single pulse energy transmitted by the fiber.

The generated plasma spectrum was coupled to several optical fibers and finally transmitted to the spectrometer produced by Avantes Ltd., which included four spectrometers with spectrum range of 180–760 nm, and typical resolution of 0.01 nm (FWHM).

The novelty of the system shown in Figure 1 is that SP-LIBS and direct-focused LA-LIBS can be realized respectively by adjusting the positions of several groups of lenses and using different lasers. In SP-LIBS, only the high single-pulse energy laser (200 mJ/pulse) is needed to work, the position of lens1 moves down, so that the focusing spot of the laser is on the surface of the soil sample, and in direct-focused LA-LIBS, fiber laser and the high single-pulse energy laser are required to work at the same time, the fiber laser is focused on the sample surface through lens2, but lens 1 needs to be moved up to focus the high-energy nanosecond pulse laser on the aerosol (about 2 mm above the ablation point of fiber laser) formed by the fiber laser ablation of the sample. Of course, the position of the spectrum collection lens needs to move up and down with the focus point of the high single-pulse energy laser to ensure that maximum plasma spectral intensity is obtained. The focal length of lens1 is 70 mm, and the focal length of lens2 is 100 mm. Whether in SP-LIBS or direct-focused LA-LIBS system, the ablation spot diameter of laser on the sample surface is 0.8 mm.

### 2.3 Experimental processes

With reference to the National Institute of Standards and Technology (NIST) database and related references, three potassium characteristic spectral lines at K I 766.45 nm, K I 769.90 nm, and K I 518.36 nm were examined, and the characteristic peak at 766.45 nm was used as the basis of the quantitative analysis model (having the best signal-to-noise ratio).

For the selection of acquisition time of laser induced breakdown spectra, the signal-to-noise ratios at the emission peaks of potassium were observed at different delay times of 0, 0.5, 1, 1.5, 2.0, or 2.5 µs, at last the delay time was finally set to 1 μs. The integration time was set to 1 ms, which is the minimum exposure time of the CCD spectrometer. Combining with previous practical experience and considering that the interference of laser-induced breakdown spectrum mainly comes from bremsstrahlung

at the initial stage of spectrum generation (<1 μs), there is little continuous background interference in the later stage of the plasma, so the integration time of 1 ms is also acceptable.

To ensure sufficient excitation power density and to reduce the influence of surface differences on the results, the excitation spot diameter of both lasers was set to 0.8 mm. The energy of the high single-pulse energy laser was set to 200 mJ/pulse, and the power of fiber laser was set to 30 W (10 kHz). In the direct-focused LA-LIBS system, the two lasers are in the state of asynchronous operation, fiber laser is just used to ablate samples, while high single-pulse energy laser is used to excite aerosol to generate plasma, so the synchronization of these two lasers is not necessary.

The moving speed of the sample is also a key factor to be considered. Taking sample17 as an example, we compared the average intensity of the characteristic spectra of potassium obtained by direct-focused LA-LIBS within 40 s at five different moving speeds: 0 mm/s, 0.5 mm/s, 1 mm/s and 1.5 mm/s and 2.0 mm/s.

The first groups of tablets were used to verify and calibrate the characteristic spectral lines of potassium. The duration of excitation was 40s. The sample5,8,10, 13, 16, 18 were selected to test the stability of the methods in different content segments, and the sample17 was selected to test the relationship between moving speed and spectral intensity.

**FIGURE 2:**
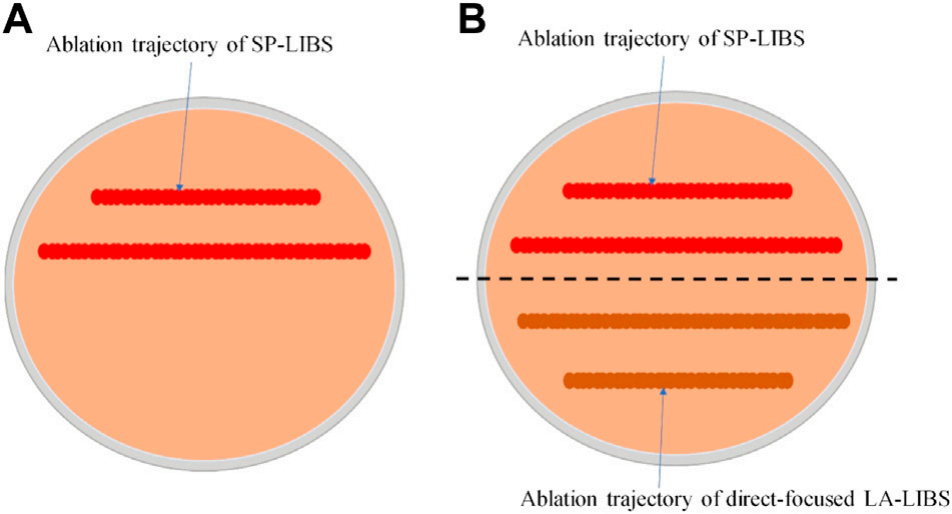
**(A)** Ablation trajectory of samples 1–3; **(B)** Ablation trajectory of samples 4–19 used to test the performance of the SP-LIBS and direct-focused LA-LIBS.

The second and third groups of tablets were used to test the performance of the SP-LIBS and direct-focused LA-LIBS methods for the analysis of potassium in soil under dry or moist conditions. and each tablet sample was divided into two excitation regions with the ablation trajectories shown in Figure 2. As in the first group, the duration of each excitation was 40 s.

## 3 Results and discussion

In this study, the spectral intensity, stability, and limit of detection were analyzed to evaluate the analytical capability of direct-focused LA-LIBS method.

### 3.1 Analysis of LIBS characteristic spectral of potassium in soil

**FIGURE 3:**
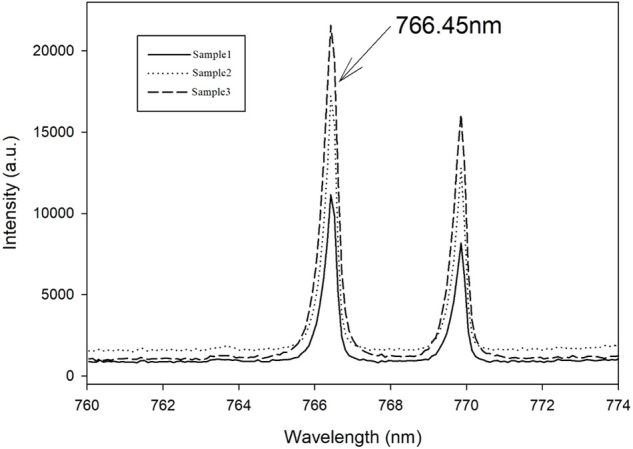
Characteristic spectrum of potassium.

The accurate determination of the characteristic peak position is the primary task of the LIBS analysis method. In our study, the characteristic spectral line at K I 766.45 nm was selected as the basis for the quantitative analysis model, as shown in Figure 3. Through the spectral analysis of the first group of tablet samples (sample1, sample2, and sample3), the characteristic spectral line of K I 766.45 nm was chosen because the best signal-to-noise ratio.

**FIGURE 4:**
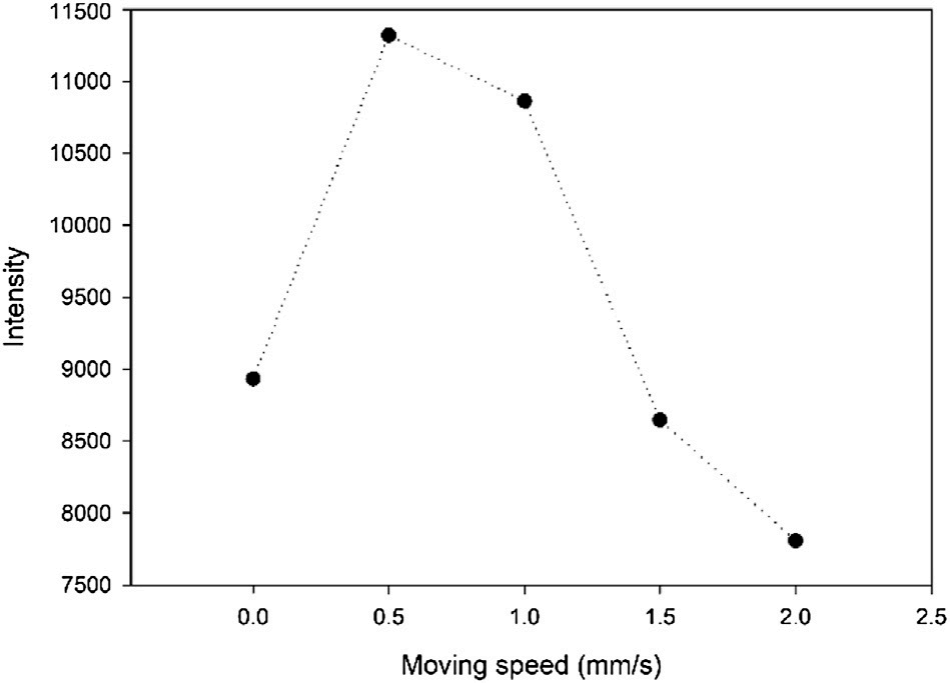
The relationship between moving speed and spectral intensity.

Some experimental parameters were set in our study, especially the moving speed of the sample is a key factor to be considered. When the speed is too fast, the fiber laser cannot effectively ablate the water and other substances in the soil, which will lead to the spectral intensity becoming low. However, if it stops, the fiber laser will form an ablation pit on the surface of the sample, which will also lead to lower spectral intensity, we compared the average intensity of the characteristic spectra of potassium obtained by direct-focused LA-LIBS within 40 s at five different moving speeds: 0 mm/s, 0.5 mm/s, 1 mm/s, 1.5 mm/s, 2.0 mm/s and the relationship between moving speed and spectral intensity is shown in Figure 4, which shows that the speed of 0.5 mm/s is appropriate.

### 3.2 Stability analysis

Signal stability is one of the most important parameters for evaluating a LIBS experimental system. In our study, relative standard deviation (RSD) was used to evaluate signal stability, which is the most important evaluation index of spectral analysis. The smaller the value of RSD, the more stable the system.

**FIGURE 5:**
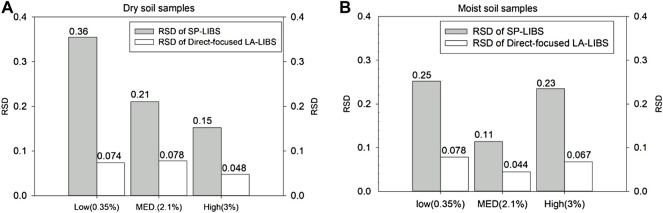
**(A)** Comparison of the RSD of SP-LIBS and direct-focused LA-LIBS for dry soil samples. **(B)** Comparison of the RSD of SP-LIBS and direct-focused LA-LIBS for moist soil samples.

As shown in Figure 5, the spectral data of three soil tablet samples with different potassium content (Low-0.35%-sample10/18, MED-2.1%-sample8/16, and High-3%-sample5/13) were selected and used to compare the signal stability of SP-LIBS and directed-focused LA-LIBS in different content segments. The results showed that the 10 kHz fiber laser ablation-assisted approach effectively improved signal stability, which further shows that it can provide enough energy to ablate samples under slow-moving conditions, and the amount of ablation is not very related to matrix differences (soil types and moisture content).

### 3.3 Limit of detection and analytical models

The limit of detection is also important index to evaluate the sensitivity of analysis model. The limit of detection in this study was evaluated by the following formula:

LOD=(3δ_background_)/K, where δ_background_ is the standard deviation of spectral signal background value, and K is the slope of calibration curve.

As shown in Table 5, Compared with the SP-LIBS system, the direct-focused LA-LIBS system had a lower LOD due to the lower standard deviation δ_background_, which also indirectly showed that the direct-focused LA-LIBS proposed has comparable or possibly higher sensitivity compared to the LA-LIBS system, which requires carrier gas.

**TABLE 5:** Comparison of LOD for SP-LIBS and direct-focused LA-LIBS.

	Parameters	Dry soil samples	Moist soil samples
SP-LIBS	3δ_background_	74.4	87.6
	LOD	9.8 μg/g	159.2 μg/g
Direct-focused LA-LIBS	3δ_background_	31.1	55.6
	LOD	7.8 μg/g	140.9 μg/g

**FIGURE 6:**
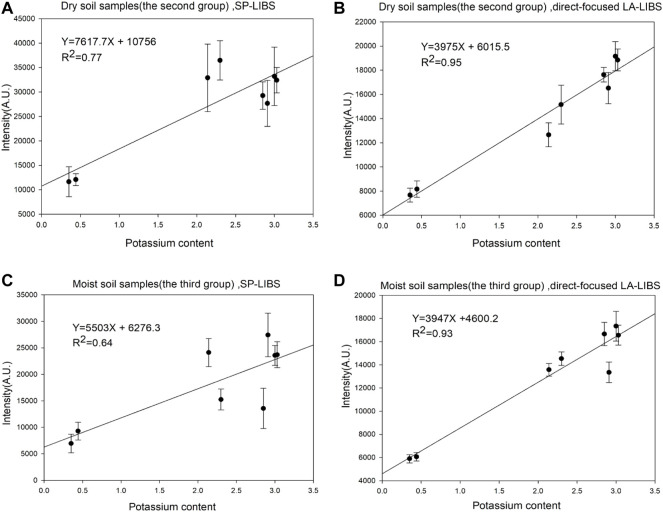
**(A)** The calibration curve of the SP-LIBS for dry soil samples. **(B)** The calibration curve of the direct-focused LA-LIBS for dry soil samples. **(C)** The calibration curve of the SP-LIBS for moist soil samples. **(D)** The calibration curve of the direct-focused LA-LIBS for moist soil samples.

As shown in Figure 6, the R^2^ factor of the calibration curves of the direct-focused LA-LIBS is 0.93 (dry soil sample) and 0.95 (moist soil sample). Compared with the SP-LIBS system, the direct-focused LA-LIBS method was less affected by the influence of soil matrix differences, especially moisture content. We believe this is due to two reasons: one, the separation of the process of laser ablation and plasma generation, effect matrix differences on laser ablation is lower under the condition of the same pulse width. Two, in the process of laser ablation and aerosol gelation of the sample, the influence of some substances on the spectrum will also be reduced. For example, when the laser focuses on the surface of the soil sample, substances with low-melting points, such as water, will be evaporated first, while the influence of water molecules at high temperatures on the spectrum will be minimized. Therefore, the direct-focused LA-LIBS system eliminates the influence of water.

Finally, we believe that different sample characteristics and experimental system parameters need to be further studied, such as the influence of laser power, focus position, and the moving speed of the mobile platform on the analysis results.

### 3.4 The LIBS analysis of field soil samples

The method modeled by the direct-focused LA-LIBS was used to analyze randomly obtained soil samples, compared with the AAS (Atomic Absorption Spectroscopy). The soil used were mainly from three areas, M1 samples was from farmland in the western suburbs of Beijing, M2 samples were from Beijing south park, and M3 samples were from Beijing YongDing riverside. The water content of the samples was M3>M1>M2, and 30 g of each sample was taken and placed in an aluminum sample box with a diameter of 40 mm, and other experimental parameters and spectral processing methods are consistent with the experiments above.

As shown in Table 6, the change in potassium detection results of the direct-focused LA-LIBS was consistent with those of AAS, indicating that the direct-focused LA-LIBS method effectively overcomes the matrix effect, especially the influence of moisture, and it does not require a carrier gas allowing for potential field application that requires rapid screening.

**TABLE 6:** Comparison of analysis results.

Sample	K (mg/g)	
	AAS	direct-focused LA-LIBS
M1	28	29.5 (±2.33)
M2	18	17.1 (±1.28)
M3	2.9	3.2 (±0.17)

The results of direct-focused LA-LIBS detection fluctuated greatly compared with traditional laboratory methods, and relative errors of direct-focused LA-LIBS detection in different content sections were 5.4, 5, and 10.3%. In future studies, improvements are needed to make the analysis results more stable and accurate through adjusting different experimental parameters.

## 4 Conclusion

In this study, a LA-LIBS analysis method based on the direct-focused of high single-pulse energy laser on the aerosol produced by ablation was proposed and compared with SP-LIBS in the analysis of soil samples.

When using direct-focused LA-LIBS for spectral analysis, it is necessary to focus the high single-pulse energy laser on the aerosol rather than the surface of the soil sample, which results in lower limit of detection due to the lower background value of the signal. In addition, the difference in soil water content has always been one of the key factors that have plagued rapid field detection. Here soil samples with different water content, the R^2^ factor of the calibration curve of the direct-focused LA-LIBS analysis method was 0.93, which is better than 0.64 for conventional SP-LIBS. These results also showed that the direct-focused LA-LIBS is an effective analytical method to reduce the influence of soil matrix, especially that of moisture. Compared with the LA-LIBS system that requires carrier gas and a sample chamber, the LA-LIBS proposed here also had a better limit of detection and stability, giving a strong potential for field detection applications.

## Data Availability

The original contributions presented in the study are included in the article/Supplementary Material, further inquiries can be directed to the corresponding author.
